# Supplementary material for: Ballou’s Ancestral Inbreeding Coefficient: Formulation and New Estimate with Higher Reliability
Source: Animals (Basel). 2024 Jun 21;14(13):1844. doi: 10.3390/ani14131844 (PMC11240364; doi:10.3390/ani14131844)
Supplement: Supplementary file 1 [file animals-14-01844-s001.zip › animals-3042403-supplementary.pdf]

Supplementary materials

1. **R script used for finding inbred ancestors and computing their genetic contributions to individual X in Figure 4.**

```
#
# R script for finding inbred ancestors and computing their genetic contributions
# to individual X in Figure 4.
#
library(ggroups)

ped <- data.frame(ID=1:27,
          SIRE=c(0,0,0,1,1,0,3,6,6,0,8,8, 9, 9, 9,0,13,16,11,14,14,0,19,19,21,25,23),
          DAM= c(0,0,0,2,2,0,4,5,5,0,7,7,10,10,10,0,12,15,17,18,18,0,20,20,22,24,26))
#
# Computing inbreeding coefficients of all individuals in pedigree, using 'inbreed'
# function in R package 'ggroups' [see reference 51 in the text].
#
inb_coeff <- inbreed(ped)
inb_coeff
#
# Computing lower triangular matrix L of genetic contributions
# based on the algorithm of Mrode (1996).
#
l_matrix <- diag(27)
for (i in 1:27) {
   for (j in 1:i-1) {
          if((ped$SIRE[i]!=0)&(ped$DAM[i]!=0)) {
             l_matrix[i,j] <- 0.5*(l_matrix[ped$SIRE[i],j]+l_matrix[ped$DAM[i],j])
          }
          else if ((ped$SIRE[i]!=0)&(ped$DAM[i]=0)) {
             l_matrix[i,j] <- 0.5*l_matrix[ped$SIRE[i],j]
          }
          else if ((ped$SIRE[i]=0)&(ped$DAM[i]!=0)) {
             l_matrix[i,j] <- 0.5*l_matrix[ped$DAM[i],j]
          }
          else {
             l_matrix[i,j] <- 0
          }
    }
}

gene_cont <- c(rep(0,27))
for (i in 1:26) {
```

```
  if(inb_coeff[i]>0) {
    gene_cont[i] <- l_matrix[27,i]
  }
}

ped2 <- cbind(ID=ped$ID,ID_farher=ped$SIRE,ID_mother=ped$DAM,gc=gene_cont)
ped2
write.table(ped2, file="habsburg_ped2.txt",row.names=FALSE,col.name=FALSE)
```

2. **Fortran code used for obtaining the results in Table 2. Prior to running this program, the input-file should be created by running the R script given above. By adding several 'write' sentences, Wright's inbreeding coefficient and Kalinowski's new and ancestral inbreeding coefficients are additionally obtained from the ordinary gene-dropping simulation.**

```
      program hybrid_simulation
!----------------------------------------------------------------------------
!       * Source code of simulation program used in section 4.
!       * Prior to running this program, the input file should be created via
!         R script given in Supplementary material 1.
!       * Wright's inbreeding coefficient and Kalinowski's ancestral inbreeding
!         coefficient from ordinary gene-dropping simulation can be obtained by
!         slight modification of this code.
!
!       n       :number of individulas in pedigree
!       ntrial:number of trial of gene dropping simulation
!       nrep    :number of rerplicates per trial (input variable given from consol)
!----------------------------------------------------------------------------
      implicit none
      integer::n,ntrial,nrep
      parameter (n=50,ntrial=100)
      integer::nn,nf,nfg
      integer::i,ii,irep,itrial
      integer::indv(n),isire(n),idam(n)
      integer::genet(1:n,2),iflag(1:n,2),sflag,dflag,jflag,kflag
      integer::ifounder(n)
      real::f_new_bal(n),f_new_kal(n),f_wright(n),f_anc_bal(n),gc(n),f_hybrid
      real::av_gds, av_hybrid
      real::rnunf
!
!       * Change the specification of files in the open statements,
!           according to the directory and folder where the files are stored.
!
      open (unit=1,file='habsburg_ped2.txt',status='unknown')
      open (unit=2,file='result_sim.txt',status='unknown')

      write(*,*) 'n of (replcates/trial)='
      read(*,*) nrep                              ! give nrep from console

      call random_seed

      nn=0
      nf=0
```

```
        do i=1,n
          ifounder(i)=0
        end do
        do i=1,n
          read(1,*,end=99) indv(i),isire(i),idam(i),gc(i)
          if(isire(i)==0.and.idam(i)==0) then
            nf=nf+1
            ifounder(i)=1
            nfg=2*(nf-1)+1
            genet(i,1)=nfg                    ! allocating first allele to a founder
            genet(i,2)=nfg+1                  ! allocating second allele to the founder
          end if
          nn=nn+1
        end do

99      write(*,*) 'number of individuals in pedigree=',nn
        write(*,*) 'number of founders=',nf

        av_gds=0.0
        av_hybrid=0.0

!************************
        do itrial=1,ntrial
!************************

        do i=1,n
          f_new_bal(i)=0.0
          f_anc_bal(i)=0.0
          f_new_kal(i)=0.0
          f_wright(i)=0.0
        end do

!------------------------
        do irep=1,nrep
!------------------------

        do i=1,nn
          iflag(i,1)=0
          iflag(i,2)=0
        end do

!==============
        do i=1,nn
!==============
          if(ifounder(i)==1) then
```

```
      goto 10
   end if

   ii=1
   call random_number(rnunf)
   if(rnunf<0.5) then
        ii=2
   end if
   genet(i,1)=genet(isire(i),ii)
   sflag=iflag(isire(i),ii)
   iflag(i,1)=sflag

   ii=1
   call random_number(rnunf)
   if(rnunf<0.5) then
        ii=2
   end if
   genet(i,2)=genet(idam(i),ii)
   dflag=iflag(idam(i),ii)
   iflag(i,2)=dflag

   kflag=sflag+dflag
   if(kflag==2) then
     f_anc_bal(i)=f_anc_bal(i)+1.0
   else if(kflag==1) then
     f_anc_bal(i)=f_anc_bal(i)+0.5
   end if

     if(genet(i,1)==genet(i,2)) then
          f_wright(i)=f_wright(i)+1.0
          jflag=sflag+dflag
          if(jflag==0) then
               f_new_bal(i)=f_new_bal(i)+1.0
               f_new_kal(i)=f_new_kal(i)+1.0
          else if(jflag==1) then
               f_new_bal(i)=f_new_bal(i)+0.5
          end if
          iflag(i,1)=1
          iflag(i,2)=1
      end if


10      continue

!=============
```

```
            end do
!==============


!----------------------
          end do
!----------------------

          do i=1,nn
             f_wright(i)=f_wright(i)/real(nrep)
             f_new_bal(i)=f_new_bal(i)/real(nrep)
             f_anc_bal(i)=f_anc_bal(i)/real(nrep)
             f_new_kal(i)=f_new_kal(i)/real(nrep)
          end do

          f_hybrid=0.0
          do i=1,nn
               if(gc(i)>0.0) then
                  f_hybrid=f_hybrid+gc(i)*f_new_bal(i)
               end if
          end do

!        * Store estimates of F_bal_anc,X from ordinary GDS and hybrid method
!           in every terial.
          write(2,'(f7.4,2x,f7.4)') f_anc_bal(nn),f_hybrid

          av_gds=av_gds+f_anc_bal(nn)
          av_hybrid=av_hybrid+f_hybrid

!********************
          end do
!********************

          av_gds=av_gds/real(ntrial)
          av_hybrid=av_hybrid/real(ntrial)

          write(*,*)
          write(*,*) 'Average of F_BAL_ANC over all trials'
          write(*,*) 'GDS-estimate      Hybrid-estimate'
          write(*,'(f7.4,2x,f7.4)') av_gds,av_hybrid

          close (1)
          close (2)

          end program hybrid_simulation
```
